# Medical Inspection of Schools

**Published:** 1907-03-30

**Authors:** 


The Hospital

A Journal of

The Medical Sciences and Hospital Administration.

Vol. XLI.—No. 1072. SATURDAY, MARCH 30, 1907.

### Medical Inspection of Schools.

One of the few clauses of last year's ill-fated Education Bill which met with general support from all parties, was that which provided for the medical inspection of school children. It, of course, came to grief with the Bill of which it formed a part. A special Bill has now been introduced to provide such inspection; it has secured a second reading, and there is every reason to believe that it will become law this session. The question will then be to consider how practical effect can be given to the law in the different parts of the country. The principle of medical inspection is not a new one, but has been recognised and adopted to a greater or less extent in several cities and counties, so that we already possess data on which to frame a more extensive organisation. There are, however, many points open to discussion in regard to the methods by which medical supervision of the schools and school children should be carried out. They may be grouped under two chief headings—firstly, the scope which the work of inspection shall assume, and, secondly, by whom the work shall be carried out.

The subject was thoroughly discussed at a recent meeting of the Medical Officers of Schools Association, and in a paper contributed by Dr. R. H. Crowley, medical superintendent of the Bradford Education Committee, the scope of the work of school inspection was clearly laid down. It should include: general advice to the Education Committee in all matters relating to the hygiene of the school and of the scholar; sanitary inspection of the school buildings in regard to ventilation, accommodation, lighting, heating, and other details; measures to check the spread of infectious diseases; separation of the physically defective children—blind, deaf, cripples—mentally defective, and epileptic; examination of all children on their first admission to school, and subsequently at regular intervals; medical examination of the teaching staff, and provision for instruction of the teachers, and also of the children, in the elements of hygiene. From this enumeration it will be seen that the scope of the work is wide, and is such as could not possibly be carried out by a single medical officer over a large county area or in a populous city. At the same time, the work will require proper organisation, and for this purpose the Education Committee must look to one medical officer to supervise, control, and analyse the work of the individual medical examiners of the schools.

In regard to this chief or head official, in certain places the Education Committee has adopted the plan of appointing its own medical officer to carry out the work. On the other hand, in other parts the work has been added to the duties of the medical officer of health for the district. There are many points in favour of this latter alternative. It is much better that there should be one central authority on such questions, than that there should be overlapping of the functions of two medical officers, and possibly consequent friction. In many cases no doubt the duties of the medical officer of health are already sufficiently onerous without any further additions, but this difficulty might be overcome by providing him with a sufficient staff of assistants, and thus an economy might be effected, as against the employment of a separate education medical officer.

Whichever alternative may be adopted, it will be quite impossible for any one official to carry out the work of inspection of the children of the individual schools in an effectual and adequate fashion. The question then arises: How are the details of the work as above laid down to be administered? In Germany and in America the work of weighing, measuring, and special examination of the children is carried out by general practitioners, who visit the schools of their districts in their daily rounds, and this would appear to be the most economical and also the most satisfactory manner of solving the problem.

Not the least important part of the scheme should be the instruction of the teachers in the elements of hygiene. By this means they would be placed in a position to be of material assistance to the medical inspector in making a preliminary selection of those scholars requiring his special attention, and they would be educated to take an intelligent interest in the health and well-being of the children under their care.

The acute problem which will eventually present itself for solution will be the treatment of the children who are found physically unfit, whether by defective vision or hearing, or by tuberculosis, or other disease. Medical inspection will point to the evils that are present; but how are they to be cured? When through the poverty or apathy of the parents no steps are taken to relieve the children, who is going to intervene? The State, having accepted the burden of providing education, now recognises the further duty of securing the child against injury to its health by providing medical inspection and free meals. Will the State also be compelled to make provision for medical treatment? That is, of course, a question for the future, and one that does not come within the scope of the present article, but medical treatment would seem to be the natural and logical corollary to medical inspection.

